# Proteomic profiling of cisplatin-resistant and cisplatin-sensitive germ cell tumour cell lines using quantitative mass spectrometry

**DOI:** 10.1007/s00345-022-03936-1

**Published:** 2022-01-27

**Authors:** A. Fichtner, H. Bohnenberger, O. Elakad, A. Richter, C. Lenz, C. Oing, P. Ströbel, S. Kueffer, D. Nettersheim, F. Bremmer

**Affiliations:** 1 Institute of Pathology, University Medical Center, Robert-Koch-Str. 40, 37075 Göttingen, Germany; 2 Department of Clinical Chemistry, University Medical Center, Göttingen, Germany; 3 Bioanalytical Mass Spectrometry Group, Max Planck Institute for Biophysical Chemistry, Göttingen, Germany; 4 Department of Oncology, Hematology and Bone Marrow Transplantation with Division of Pneumology, University Medical Center Hamburg-Eppendorf, Martinistraße 52, 20246 Hamburg, Germany; 5 Mildred Scheel Cancer Career Center HaTriCS4, University Medical Center Eppendorf, Hamburg, Germany; 6 Department of Urology, Urological Research Laboratory, Translational UroOncology, Medical Faculty and University Hospital Düsseldorf, Heinrich Heine University Düsseldorf, Universitätsstrasse 1, 40225 Düsseldorf, Germany

**Keywords:** Germ cell tumour, Cisplatin resistance, Proteomic analysis, Mass spectrometry, SILAC

## Abstract

**Purpose:**

Advanced testicular germ cell tumours (GCT) generally have a good prognosis owing to their unique sensitivity towards cisplatin-based chemotherapies. However, cisplatin-resistant GCT have a poor outcome. Further studies are mandatory to better understand resistance mechanisms and develop therapeutic strategies for refractory GCTs.

**Methods:**

Protein levels in cisplatin-resistant GCT cell lines of NTERA-2, NCCIT and 2102EP were analyzed by quantitative proteomic mass spectrometry (MS) in combination with stable isotope labelling by amino acids in cell culture (SILAC). Differentially abundant protein markers of acquired cisplatin resistance were validated by Western blotting. Comprehensive bioinformatical annotation using gene set enrichment analyses (GSEA) and STRING interaction analysis were performed to identify commonly affected pathways in cisplatin resistance and the data were compared to the GCT cohort of the ‘The Cancer Genome Atlas’.

**Results:**

A total of 4375 proteins were quantified by MS, 144 of which were found to be differentially abundant between isogenic resistant and sensitive cell line pairs (24 proteins for NTERA-2, 60 proteins for NCCIT, 75 proteins for 2102EP). Western blotting confirmed regulation of key resistance-associated proteins (CBS, ANXA1, LDHA, CTH, FDXR). GSEA revealed a statistically significant enrichment of DNA repair-associated proteins in all three resistant cell lines and specific additional processes for individual cell lines.

**Conclusion:**

High resolution MS combined with SILAC is a powerful tool and 144 significantly deregulated proteins were found in cisplatin-resistant GCT cell lines. Our study provides the largest proteomic in vitro library for cisplatin resistance in GCT, yet, enabling further studies to develop new treatment options for patients with refractory GCT.

**Supplementary Information:**

The online version contains supplementary material available at 10.1007/s00345-022-03936-1.

## Introduction

Testicular germ cell tumours (GCTs) are the most common malignant solid neoplasms in men between the age of 15 and 40 with an increasing incidence seen over the last 4 decades [1]. Histologically, type II GCT are divided into seminomas (SEM) and nonseminomatous GCT (NSGCT) according to the WHO [2]. NSGCT comprise distinct subentities, e.g. embryonal carcinomas (EC), yolk sac tumours, choriocarcinomas and teratomas (TER) [3]. The distinction between SEM and NSGCT is important because of varying treatment approaches, treatment responses and patients’ prognosis [4]. The treatment of GCTs primarily comprises a radical inguinal orchiectomy of the affected testis. Subsequent cisplatin-based combination chemotherapy is required in metastatic GCTs [5, 6]. The introduction of cisplatin-based combination chemotherapy has led to cure rates of up to 90% even in metastatic disease stages [7–9]. Teratomas are of particular interest, as they are uniformly cisplatin-resistant and surgery is the only successful therapy [6]. The number of patients with recurring disease that finally fail several lines of platinum-based chemotherapy is about 3–5% of all GCT patients and about 15% of patients with primary metastatic disease [5, 8, 10, 11], but they have an exceptionally poor prognosis with a life expectancy of only a few months [12].

In this study, we compared cisplatin-resistant and cisplatin-sensitive cell lineages of pluripotent NTERA-2 and NCCIT as well as the nullipotent 2102EP cells (with NCCIT deriving from a TP53 mutated mixed mediastinal GCT) [13, 14] by high-resolution mass spectrometry (MS) combined with stable isotope labelling with amino acids in cell culture (SILAC) [15–18]. The NTERA-2 and NCCIT cell lines represent EC, which can develop in all other types of NSGCT. Both cell lines represent a very well-studied in vitro model for NSGCTs. 2102EP represents an undifferentiated nullipotent EC, i.e. expresses markers of pluripotency, but do not tend to differentiate in response to differentiation-inducing signals. Cell lines representing seminoma were not used. We provide a proteomic resource library and functional annotations determined by gene set enrichment analyses (GSEA), the STRING algorithm and DAVID Gene Ontology annotation of acquired cisplatin resistance in GCT cell lines and compared our findings to the ‘The Cancer Genome Atlas’ (TCGA) cohort of ‘testicular germ cell tumours’. These findings may help to detect new treatment options for GCT patients with cisplatin-resistant disease course. There are already some treatment options available, but as common mutations (e.g. receptors, kinases, etc.) are lacking in GCT, targetable mechanisms of cisplatin resistance need to be discovered and further analysed.

## Material and methods

### Culture of human GCT cell lines

The two human GCT cell lines NTERA-2 (CRL 1973) and NCCIT (CRL 2073) were supplied by ATCC, USA. 2102EP cells were a kind gift from PD Dr. Dr. Friedemann Honecker. All cell lines were cultured in HEPES-buffered RPMI-1640 (Biochrom, Berlin, Germany) supplemented with fetal calf serum (FCS, 10%; CC Pro, Neustadt, Germany), penicillin (100 IU/ml; Sigma-Aldrich, Munich, Germany), streptomycin (100 μg/ml; Sigma-Aldrich) and l-glutamine (2 mM; Biochrom, Berlin, Germany). The incubation temperature was 37 °C in a humid atmosphere with 5% carbon dioxide in the air.

### Induction of cisplatin resistance in human GCT cell lines

**Fig. 1:**
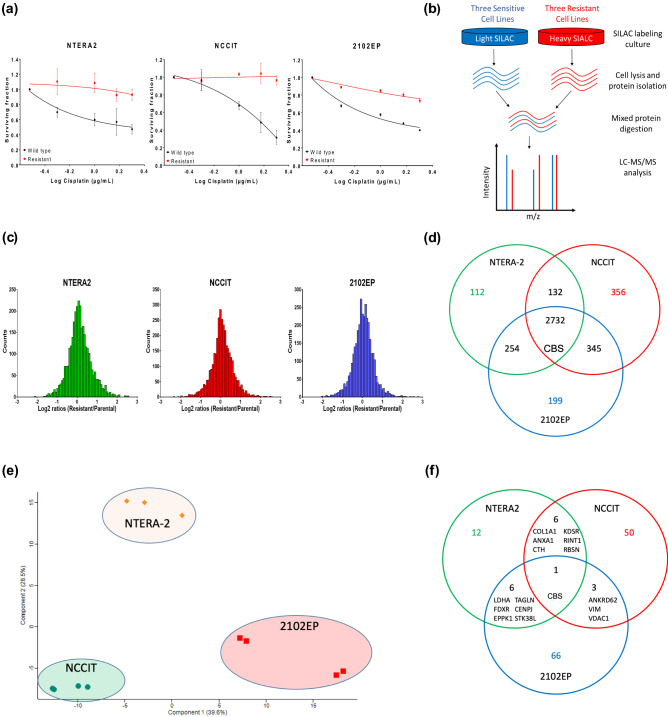
Proteomic comparison of cisplatin-resistant and cisplatin-sensitive GCT cell lines. **a** Comparison of cellular viability with increasing cisplatin concentrations in cultures of cisplatin-sensitive and cisplatin-resistant cell lines of NTERA-2, NCCIT and 2102EP with significant higher viability in resistant cell lines with high concentrations of cisplatin. **b** The diagram demonstrates the workflow of the three cisplatin-resistant and three cisplatin-sensitive cell lines for SILAC labelling and LC–MS/MS analysis. **c** Normal distribution of log_2_ SILAC ratios among the three named cell lines was seen. **d** Numeric Venn diagram of all quantified proteins in the three cell lines NTERA-2, NCCIT and 2102EP. **e** Principle component analysis showing technical and biological reproducibility of proteomic analysis **f** Numeric Venn diagram of significantly differentially regulated proteins in all three cell lines

For all parental GCT cell lines (NTERA-2, NCCIT, 2102EP), isogenic cisplatin-resistant sublines (NTERA-R, NCCIT-R, 2102EP-R) were established through repeated cisplatin-exposure to increasing sublethal cisplatin concentrations (0.01–0.5 µg/ml) over a time period of 9–12 months, as described previously [19]. Cisplatin resistance was validated by treating sensitive and resistant cell lines after 72 h with increasing concentrations of cisplatin followed by comparing cellular viability using the CellTiter 96 R AQ One Solution Cell Proliferation Assay (Promega GmbH, Waldorf, Germany) (Fig. 1a).

### Proteomic analysis of human GCT cell lines

We performed protein expression profiling by mass spectrometry in combination with stable isotope labelling by amino acids in cell culture (SILAC) as described before [15–18]. NTERA-2, NCCIT and 2102EP cells and their resistant counterparts NTERA-2-R, NCCIT-R and 2102EP-R were cultured in RPMI 1640 medium supplemented with 10% dialyzed FCS (Invitrogen, Thermo Fisher GmbH, Bremen, Germany), 4 mM glutamine, antibiotics and 0.115 mM l-arginine-^13^C_6_ (Arg + 6) and 0.275 mM l-lysine-^2^D_4_ (Lys + 4) or equimolar amounts of l-arginine-^13^C_6_^15^N_4_ (Arg + 10) and 0.275 mM l-lysine-^13^C_6_,^15^N_2_-Lys (Lys + 8) (Eurisotop GmbH, Saarbrücken, Germany) for at least ten cell cycles. Labelled cells were lysed in 0.5% Nonidet P-40 buffer containing 50 mM Tris/HCl, pH 7.8, 150 mM NaCl, 1 mM Na_3_VO_4_, 1 mM NaF, 0.2% lauryl maltoside and protease inhibitors (cOmplete™ Protease Inhibitor Cocktail, Roche, Mannheim, Germany). After isolation, the protein amounts were determined by a colorimetric BCA assay. Equal amounts of SILAC-labelled proteins (50 µg for each trial), were mixed and separated by SDS-PAGE (4–12% NuPAGE Bis–Tris Gel, Invitrogen, Thermo Fisher Scientific GmbH, Germany). Proteins were visualized with Coomassie brilliant blue stain and each lane cut into 11 equidistant slices irrespective of staining. Gel slices were reduced with 10 mM DTT for 55 min at 56 °C, alkylated with 55 mM IAA for 20 min at 26 °C and digested with modified trypsin (Promega GmbH, Walldorf, Germany) overnight at 37 °C. Resulting peptides were concentrated on a C18 precolumn (25 mm × 150 μm I.D., Reprosil-Pur C18-AQ 120 Å 5 μm, Dr. Maisch HPLC GmbH, Ammerbuch, Germany) for 5 min at a flow rate of 10 μl/min and separated on a C18 capillary column (200 mm × 75 μm I.D., Reprosil-Pur C18-AQ 120 Å 3 μm, Dr. Maisch HPLC GmbH, Ammerbuch, Germany) at a flow rate of 300 nl/min, with a gradient of 5–35% acetonitrile 0.1% formic acid over 90 min using a Proxeon nano LC coupled to a Q Exactive mass spectrometer (Thermo Fisher Scientific GmbH, Bremen, Germany). MS conditions were as follows: spray voltage, 1.8 kV; heated capillary temperature, 270 °C; and normalized collision-energy (NCE) 28. The mass spectrometer was operated in data-dependent acquisition mode. Survey MS spectra were acquired in the Orbitrap (*m/z* 350–1600) with a resolution setting of 70,000 at *m/z* 200, a target fill value of 1e6 and a maximum fill time of 60 ms. The 15 most intense ions were sequentially isolated for HCD MS/MS fragmentation and detection at a resolution setting of 17,500, a target fill value of 2e5 and a maximum fill time of 60 ms. Raw data were analysed with MaxQuant software version 1.3.0.5 (Max Planck Institute for Biochemistry, Martinsried, Germany) against the UniprotKB human reference proteome revision 02–2017 containing 92,928 sequence entries. Up to two missed cleavages of trypsin were allowed. Oxidized methionine, N-terminal protein acetylation and the respective isotope-labelled arginine and lysine residues were searched as variable modifications and cysteine carbamidomethylation as fixed modification. The false discovery rates at the protein and peptide levels were both set to 1%, respectively. Missing values in individual experiments are denoted by ‘Not a Number (NaN)’. No further data imputation or similar was used.

### Statistical analysis

Perseus Software version 1.5.2.6 (Max Planck Institute for Biochemistry, Martinsried, Germany) was used for statistical evaluation. The mean of the biological replicates was calculated for each cell line (mean resistant/parental ratio). In order to compare Log_2_ ratios of quantification to the intensity, Log_10_ values were calculated for intensities of each mean value. Afterwards, significance B analysis using a Wilcoxon-Mann–Whitney test with a Benjamini–Hochberg FDR < 5% was performed by Perseus software to calculate the *p* values of outlier proteins from the 1/1 ratio [20]. Log_10_ intensity was plotted against Log_2_ ratios of quantified resistant/parental ratios. Proteins were coloured according to their *p* values where blue colour means > 0.05, red color 0.05–0.01, yellow between 0.01 and 0.001 and light green coloured proteins mean that they have *p* values less than 0.001.

### Western blot analysis

Cell lines were lysed in RIPA buffer (1 l PBS Dulbecco pH 7.4; 5 g 5% sodium deoxycholate, 10 ml IGEPAL^®^ CA-630) with protease inhibitors. Protein concentration was quantified by the Bio-Rad DC Protein Assay (Bio-Rad Laboratories GmbH, Feldkirchen, Germany). The Western blot analyses were performed using the following primary antibody dilutions: monoclonal rabbit anti-CBS (D8F2P, Cell Signalling Technology^®^, Massachusetts, USA, 1:1000), polyclonal rabbit anti-LDHA (Cell Signalling Technology^®^, Massachusetts, USA, 1:1000), monoclonal rabbit anti-ANXA1 (D16A10, Cell Signalling Technology^®^, Massachusetts, USA, 1:1000), monoclonal rabbit anti-CTH (D4E9J, Cell Signalling Technology^®^, Massachusetts, USA, 1:1000) monoclonal mouse anti FDXR (6C2, Invitrogen, Thermo Fisher Scientific, Darmstadt, Germany 1:1000). Polyclonal immunoglobulins/HRP secondary antibodies (1:1000, Dako, Agilent Technologies GmbH, Waldbronn, Germany) were used for the detection of primary antibodies. Membranes were developed using the ECL system (Amersham Bioscience Europe GmbH, Freiburg, Germany).

### Gene set enrichment analysis

Functional annotations were predicted using gene set enrichment analysis (GSEA) software downloaded from the home page (http://www.gsea-msigdb.org/gsea/index.jsp) [21, 22]. Log2 transformed proteomics resistance vs native ratios and the value 0 as native pendant were subjected uploaded into the software. Enrichment analysis was performed on the HALLMARK GeneSets with 1000 permutations on the original dataset format and gene set as Permutation type. Significant enrichments were defined as nominal *p* values < 0.05 and FDR *q* < 25% as suggested by GSEA. For visualization in a bar graph *p*- and *q*-values were − log2 transformed. Enrichment plots were saved from the GSEA reports.

### Online analyses tools and software

The STRING algorithm was used to predict protein–protein interaction by confidence using standard settings (https://string-db.org) [23]. The DAVID annotation has been used to predict molecular functions of deregulated genes or proteins found in mass spectrometry analyses based on ‘Gene Ontology’ (GO), ‘Kyoto Encyclopedia of Genes and Genomes’ (KEGG), Uniprot and INTEPRO (https://david.ncifcrf.gov/home.jsp) [24]. The proteins detected in this study were also compared with the TCGA dataset of ‘testicular germ cell tumours’ via cBioPortal (https://www.cbioportal.org/) [25, 26].

## Results

### Quantitative proteomic profiling of cisplatin-resistant human GCT cell lines

We compared the protein profiles of three cisplatin-sensitive human GCT cell lines to their complementary cisplatin-resistant subclones using high resolution mass spectrometry in combination with SILAC (Fig. 1b). Normal Gaussian distribution of log_2_ SILAC ratios were seen in all three cell lines (Fig. 1c, Supplementary Fig. 1). In total, 4375 proteins were detected (Fig. 1d, Supplementary Table 1). Principle component analysis demonstrated high technical and biological reproducibility of proteomic analysis (Fig. 1e). A significantly different regulation between resistant and sensitive cell lines was found for 25 proteins in NTERA-2, for 60 proteins in NCCIT and for 75 proteins in 2102EP (Fig. 1e and f, 2a–c). Of these, 16 were significantly deregulated in at least two of the three tested cell lines (Table 1).

**Fig. 2:**
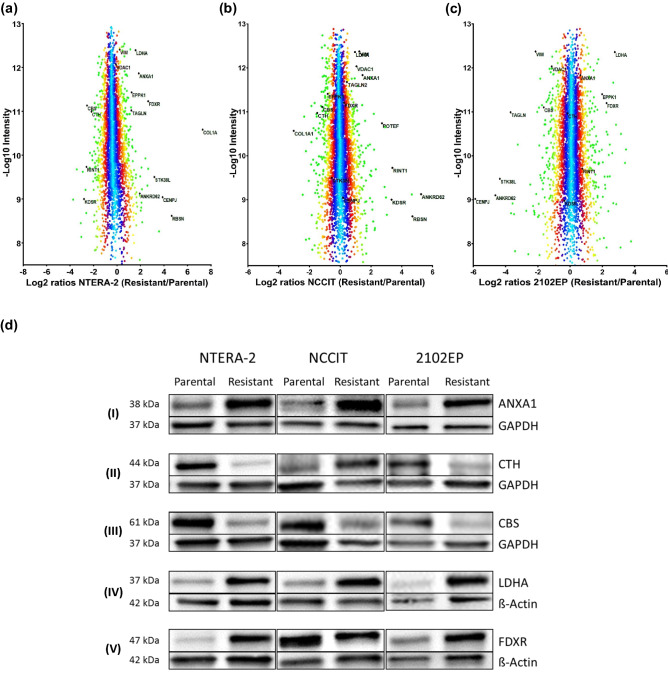
Distribution of proteins from SILAC and validation of protein expression by Western Blot analysis. **a**–**c** Distribution of SILAC ratios of all quantified proteins according to their relative expression in the resistant vs. sensitive cells lines of NTERA-2, NCCIT and 2102EP. **d** CBS (**I**) is downregulated in NTERA-2-R, NCCIT-R and 2102EP-R. ANXA1 (**II**) and LDHA (**III**) are upregulated in NTERA-2-R, NCCIT-R and 2102EP-R. FDXR (**IV**) is upregulated in NTERA-2-R and 2102EP-R with similar expression in NCCIT and NCCIT-R. CTH (**V**) is downregulated in NTERA-2-R and 2102EP and shows higher expression in NCCIT-R

**Table 1:** Overlapping proteins possibly associated with cisplatin resistance

Protein name	Gene name	NTERA-2 (R/S)	*p* value	NCCIT (R/S)	*p* value	2102EP (R/S)	*p* value
Cystathionine beta-synthase	CBS	0.24 ↓	2.38E-06	0.44 ↓	4.52E-04	0.34 ↓	2.20E-04
Annexin A1	ANXA1	4.89 ↑	3.13E-10	3.14 ↑	2.46E-06	1.44 ↑	1.98E-01
Rabenosyn-5	RBSN	34.36 ↑	3.16E-09	35.74 ↑	1.78E-14	NaN	NaN
l-lactate dehydrogenase A chain	LDHA	4.10 ↑	2.29E-08	2.18 ↑	1.35E-03	5.47 ↑	5.79E-10
NADPH:adrenodoxin oxidoreductase, mitochondrial	FDXR	8.59 ↑	3.18E-13	1.27 ↑	4.72E-01	3.96 ↑	1.63E-07
Epiplakin	EPPK1	3.24 ↑	3.48E-06	0.58 ↓	1.19E-02	3.31 ↑	1.33E-05
Ankyrin repeat domain-containing protein 62	ANKRD62	5.22 ↑	9.97E-03	53.86 ↑	1.51E-18	0.05 ↓	2.41E-18
Vimentin	VIM	1.60 ↑	6.77E-02	2.70 ↑	4.32E-05	0.25 ↓	5.46E-10
Voltage-dependent anion-selective channel protein 1	VDAC1	1.30 ↑	3.26E-01	2.34 ↑	4.78E-04	0.47 ↓	6.49E-04
Collagen alpha-1(I) chain	COL1A1	210.43 ↑	1.37E-56	0.11 ↓	3.20E-12	NaN	NaN
Cystathionine gamma-lyase	CTH	0.31 ↓	1.02E-04	0.35 ↓	7.14E-06	0.87 ↓	5.87E-01
3-ketodihydrosphingosine reductase	KDSR	0.20 ↓	2.02E-04	12.86 ↑	2.25E-08	0.77 ↓	5.43E-01
RAD50-interacting protein 1	RINT1	0.23 ↓	6.19E-05	13.42 ↑	1.29E-10	1.55 ↑	1.84E-01
Transgelin	TAGLN	3.20 ↑	1.12E-04	NaN	NaN	0.09 ↓	7.36E-16
Centromere protein J	CENPJ	19.94 ↑	7.19E-07	1.21 ↑	7.47E-01	0.02 ↓	2.21E-28
Serine/threonine-protein kinase 38-like	STK38L	12.34 ↑	1.06E-06	0.70 ↓	3.04E-01	0.06 ↓	2.05E-16

The table summarizes the protein ratios in cisplatin-resistant cell lines (R) compared with the sensitive parental control cell lines (S) and the corresponding *p* values of all proteins that showed significantly different levels in at least two of the three tested cell lines. Increased levels are is highlighted with a green arrow (↑) and decreased levels are marked with a red arrow (↓)

*NaN* not a number

### Western blot analysis

We were able to confirm the obtained MS results using Western blotting of selected proteins described in Table 1 (see Fig. 2d). The selected proteins showed a significant different regulation in at least two or all three cell lines in comparison to the parental lineages (Table 1). All three cisplatin-resistant cell lines showed lower expression of cystathionine beta-synthase (CBS) and cystathionine gamma-lyase (CTH) levels were lower in NTERA-2-R and 2102EP-R compared with the cisplatin-sensitive counterpart (Fig. 2d I and II). Amounts of Annexin A1 (ANXA1), the l-lactate dehydrogenase A (LDHA) and NADPH-adrenodoxin oxidoreductase (FDXR) were increased in all three resistant cell lines (Fig. 2d III–V), which is in accordance with the mass spectrometric analysis results (Fig. 2a–c).

### Gene set enrichment analysis

**Fig. 3:**
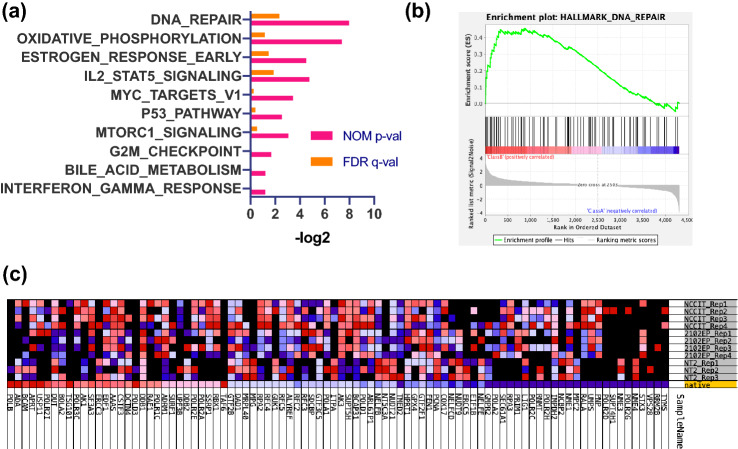
GSEA analysis reveals DNA repair as common gene set deregulated in cisplatin-resistant cells. **a** The ten most affected gene sets of the combined ratios of three NT2, four NCCIT and four 2102EP replicates of proteomic analysis represented as the − log2 of the nominal (NOM) *q* value and the FDR *q* value. **b** Enrichment plot: DNA REPAIR Profile of the Running ES Score and Positions of GeneSet Members on the Rank Ordered List (*p* < 0.004, *q* < 0.19). **c** Blue–Pink O’ Gram in the Space of the analyzed GeneSet representing the ratio values of all genes in the GeneSet referred to 0

In order to gain functional insights into the mechanisms of cisplatin resistance, we performed a gene set enrichment analysis (GSEA) of all genes coding for proteins altered in each cell line individually or commonly. GSEA determines, if a defined set of genes shows a statistically significant difference in enrichment between two biological states (cisplatin resistance vs. sensitivity) [21, 22]. We compared the cisplatin-resistance ratios to 50 hallmark gene sets representing specific biological states or processes (http://www.gsea-msigdb.org/gsea/msigdb/collections.jsp). In each cisplatin-resistant cell line individually, we found a heterogenous set of processes affected. The analysis of NTERA-2-R detected ‘Interferon alpha and gamma’ signalling and ‘epithelial to mesenchymal transition’ (EMT) as mainly enriched gene sets (Supplementary Fig. 2a–d). Two sets related to ‘Myc targeting’ and ‘DNA repair’ were significantly affected in NCCIT-R cell line (Supplementary Fig. 2e–h). Furthermore, GSEA revealed seven significantly affected gene sets including ‘P53 signalling’, ‘hypoxia’, ‘fatty acid metabolism’, ‘glycolysis’, ‘late estrogen response’, ‘oxidative phosphorylation’ and ‘IL2 STAT5 signalling’ for 2102EP-R (Supplementary Fig. 2i–p). The combined analysis of the three cell lines highlighted ‘DNA repair’, ‘oxidative phosphorylation’ and ‘early estrogen response’ as the three most prominently enriched gene sets (Fig. 3a–c).

### STRING interaction prediction, DAVID gene ontology and TCGA cohort analysis

First, the DAVID Gene ontology tool was used to predict molecular functions of all proteins altered in the resistant cell lines (Supplementary Fig. 3 a, c, e, g, i, k). Proteins increased in amount in the resistant cells compared with the parental cell lines were involved in oxidoreductase activity, acetylation and metal binding were among others commonly deregulated in all three cell lines. Next, the STRING algorithm was used to predict protein–protein interactions of the enriched proteins (Supplementary Fig. 3b, d, f). Proteins involved in the molecular processes found by the DAVID analysis were highlighted by colour. In contrast, proteins decreased in amount in resistant cell lines compared with the parental cell lines were involved in phosphatidyl-inositol binding and DNA methyltransferase activity (Supplementary Fig. 3 g–l).

Furthermore, using cBioPortal, we screened the TCGA GCT tissue cohort for mutational footprints and expression of all genes coding for affected proteins in resistant cell lines individually (Supplementary Fig. 4). In general, 149 GCT (seminoma and nonseminoma) samples were included. The majority of the samples were highly aneuploid, harbored a 12gain, were mainly in the typical age of 14–44 years and were isolated from testis. Indeed, expression of analyzed genes was mainly associated with a nonseminomatous cell character (Supplementary Fig. 4). Nevertheless, expression of a subset of genes could also be related to seminomas. Mutations in the analyzed genes were overall rare, with *IFITM3* (enriched in 2102EP-R) harboring a deep deletion in 2.1% of samples, *GDF3* being amplified in 7% (enriched in NCCIT-R) and *STK38L* (enriched in NT2/D1-R) being amplified in 8% as most affected genes by mutation.

## Discussion

Cisplatin resistance in GCTs rarely occurs in primary diseases, but in up to 15% of metastatic diseases with poor prognosis for the affected patient [5]. The mechanisms of cisplatin resistance in GCTs are complex, still not completely understood and might depend on GCT subtypes [27]. Different cellular and molecular mechanisms and GCT characteristics, such as DNA damage repair systems, the p53/MDM2 axis and apoptotic pathways as well as epigenetic changes have been investigated and seem to contribute to cisplatin resistance as a multifactorial phenomenon [27]. Because tissue specimens from cisplatin-resistant GCTs are rare, we analyzed the protein expression profiles of three different cisplatin-resistant GCT cell lines (NTERA-2-R, NCCIT-R and 2102EP-R) and their cisplatin-sensitive parental counterparts using mass spectrometry in combination with stable isotope labelling with amino acids in cell culture (SILAC) [13, 14] to detect proteins with a possible impact on cisplatin resistance. In addition, GSEA, DAVID Gene Ontology and STRING analyses were performed and the results were compared with the TCGA GCT tissue cohort.

Cystathionine beta-synthase (CBS) was significantly decreased in amount in all three cisplatin-resistant cell lines in comparison to their cisplatin-sensitive parental lineage. CBS is an enzyme that regulates homocysteine metabolism and catalyzes the formation of cystathionine [28]. Furthermore, it participates in different desulfurization reactions, which lead to the production of hydrogen sulfide (H_2_S) [29]. CBS is most commonly synthesized in the liver, the pancreas, the kidney and the brain with only low baseline expression in testicular tissues [30, 31]. Its role in cancer biology is complex and seems to be cancer type-specific [30]. For example, serous ovarian cancer [32] as well as invasive urothelial bladder carcinoma [33], colorectal cancers [27] and prostate cancer [28] have shown high levels of CBS and inhibiting CBS has improved the effect of cisplatin-based chemotherapy in these neoplasms. The downregulation of CBS via small molecule inhibitors or siRNAs reduced antioxidant capacity and therefore enhanced the sensitivity of cancer cells to chemotherapy. It is also suggested that decreased levels of CBS and H_2_S production might activate the intrinsic apoptotic pathway via release of mitochondrial cytochrom C [34]. According to the human protein atlas [35], testicular GCT tissue show low synthesis of CBS and other molecules of sulfur metabolism (e.g. glutathione (GSH) or methallothioneins). Masters et al. also showed, that cisplatin-sensitive GCT cell lines have low levels of GSH and glutathione-*S*-transferase (GST) [36]. These findings are not quite in accordance with our findings of low CBS levels in cisplatin-resistant GCT cell lines, but further investigations are required to better define its role in resistance.

Another enzyme of the cysteine metabolism, namely cystathionine gamma lyase (CTH), showed decreased levels in two of the three resistant cell lines (NTERA-2-R and NCCIT-R). CTH catalyzes the breakdown of cystathionine to cysteine, alpha-ketobutyrate and H_2_S. It plays a key role in the bodies H_2_S production [28, 30]. Its function in tumour biology has only been investigated in some tumour entities. The studies showed, similar to the role of CBS in the abovementioned tumours, that murine prostate cancer cells showed high amounts of CTH and its product H_2_S in metastatic prostate cancer [37], similar to breast cancer cells [38, 39]. Inhibition of CTH resulted in a decreased tumour burden. In our study, resistant NTERA-2 and NCCIT showed decreased levels of CTH in comparison to the sensitive cell lines. A function of low CTH levels in resistant tumour cell lines remains to be elucidated, yet.

The Annexin A1 (ANXA1) levels were significantly increased in resistant NTERA-2 and NCCIT cell lines. ANXA1 is a calcium-dependent phospholipid binding protein that belongs to the Annexin superfamily [40–42]. ANXA1 is a substrate for different kinases, e.g. epidermal growth factor receptor kinase, and is involved in different cellular pathways which are in association with inflammation, cell differentiation and proliferation [43]. Its role in tumour biology (e.g. tumour development, proliferation) has been conflicting because its expression is increased in some cancers (e.g. esophageal, gastric, colorectal, pancreatic and lung adenocarcinoma) and decreased in others (e.g. esophageal, lung squamous cell carcinoma, breast carcinoma and prostatic adenocarcinoma), but a common function of ANXA1 in chemosensitivity was reported [44–46]. Wang et al. could show an increased ANXA1 expression in a platin-resistant cell line of pulmonary adenocarcinoma and verified this in primary tumour tissue of cisplatin-resistant patients [47]. These previously reported roles of ANXA1 complement our findings and supports the contribution of ANXA1 to the development of cisplatin resistance irrespective of the tumours’ tissues of origin.

In addition, the protein levels of L-lactate dehydrogenase A chain (LDHA) were elevated in NTERA-2-R and 2102EP-R cells. LDHA plays an important role in anaerobic glycolysis [48, 49]. In tumour cells, LDHA plays essential role in initiation, growth, tumour maintenance, progression and metastasis [49]. Increased LDH levels are often used as diagnostic markers, prognostic factors and indicators of treatment response for GCTs and other tumours [50]. According to cisplatin treatment response, Manerba et al. showed that inhibition of LDH in Burkitt’s lymphoma cells increased cisplatin sensitivity possibly through higher amounts of reactive oxygen species (ROS) [51]. This can on the opposite indicate that higher levels of LDHA in cisplatin-resistant GCT cell lines help to overcome cell stress. However, further investigations regarding the detailed role of LDHA in cisplatin-resistant cell lines are necessary.

Furthermore, a significantly increase of COL1A1 and COL1A2 levels was detected in cisplatin-resistant NTERA-2 cell lines in comparison with the parental cell line, with decrease in resistant NCCIT cell line. Both proteins resemble extracellular matrix proteins for that increased levels have been detected in stressed cells [52]. They have further been shown to play an important role in cisplatin-resistance in pulmonary adenocarcinoma cell lines [52]. It remains unclear why the protein levels are different in the three different cell lines and needs further investigation.

GSEA revealed that DNA repair is the only significant gene set deregulated in all three cell lines, which has been considered for cisplatin resistance in GCT before [27]. In NTERA-2-R, interferon alpha and gamma signalling as well as epithelial to mesenchymal transition (EMT) were the most enriched gene sets. The role of EMT in chemoresistance has been described by Ashrafizadeh et al. but has not been evaluated for GCTs [53]. NCCIT cell lines showed significantly deregulated sets of MYC targeting and DNA repair. The function of DNA repair mechanisms in GCT cisplatin resistance are still slightly controversial, since both, upregulation and downregulation of DNA repair-associated genes has been described in GCTs [27]. For 2102EP cells, GSEA revealed seven significantly affected gene sets, including p53 signalling, oxidative phosphorylation and IL2 STAT5 signalling. Mutations and inactivation of p53 are known to be involved in cisplatin resistance of GCT by reducing apoptotic cell death [54].

DAVID Gene Ontology and STRING analyses showed a deregulation of proteins involved in oxidoreductase activity, acetylation and metal binding. The upregulation of NADPH-adrenodoxin oxidoreductase (FDXR) in resistant cell lines could be validated by Western blot. Oxidoreductase activity, acetylation and metal binding have been described in connection to p53 [55–57]. A reduced p53 activity has been shown to induce an increased resistance against cisplatin in testicular germ cell-derived human embryonal carcinoma cells also by a direct regulation of FDRX [58]. Although p53 pathway was only detected as significantly regulated in cisplatin-resistant 2102EP, these data indicate a strong p53 dependency and a deregulated oxidative stress response in cisplatin-resistant GCT.

Screening of the TCGA GCT cohort demonstrated that most genes/proteins found increased in the resistant situation, show already basal expression in GCT tissues (mostly nonseminomas). Thus, rather an overshooting/upregulation of expression than an induction of gene expression is associated with acquisition of therapy resistance. The mutational burden was overall low, nevertheless some genes were frequently mutated in GCT tissues, putatively affecting their molecular function. During development of cisplatin resistance, these mutated genes might be induced as well, further contributing to cisplatin resistance by their altered function or increased expression turnover.

In summary, high resolution mass spectrometry in combination with SILAC quantification is a powerful tool to detect differences of protein levels in cisplatin-resistant and cisplatin-sensitive cell lines. We detected 144 significantly deregulated proteins were found in cisplatin-resistant GCT cell lines. The findings of mass spectrometry could be validated by Western blot analysis. With this study, we therefore provide a large proteomic resource in vitro library for studying proteomic alterations contributing to acquired cisplatin resistance in testicular germ cell tumour cell lines. The detected and analysed proteins need further investigations to unravel their putative role in cisplatin resistance of GCT and to determine possible new treatment approaches.

## Supplementary Information

Below is the link to the electronic supplementary material.

Supplementary file1 (PPTX 277 KB) Supplementary Figure 1: Distribution of proteins in all cell lines. This figure shows the normal distribution of proteins from SILAC analysis in all cisplatin-resistant and cisplatin-sensitive cell lines.

Supplementary file2 (XLSX 695 KB)

Supplementary file3 (PPTX 1041 KB) Supplementary Figure 2: GSEA analysis of all three GCT cell lines separated. (a) The top ten affected gene sets of NTERA-2 represented as the − log2 of the NOM q-value and the FDR q value. Significant enrichment plots of the resistant vs native ratios of NT2: (b) INTERFERON ALPHA RESPONSE (p < 0.001, q < 0.045), (c) INTERFERON GAMMA RESPONSE (p < 0.016, q < 0.145) and (d) MESENCHYMAL TRANSITION (p < 0.042, q < 0.224). (e) The top ten affected gene sets of NCCIT represented as the − log2 of the NOM q value and the FDR q value. Significant enrichment plots of the resistant vs native ratios of NCCIT: (f) MYC TARGETS_V1 (p < 0.000001, q < 0.014), (g) DNA REPAIR (p < 0.001, q < 0.096) and (h) MYC TARGETS V2 (p < 0.016, q < 0.111). (i) The ten top affected gene sets of 2102EP represented as the − log2 of the NOM q value and the FDR q value. Significant enrichment plots of the resistant vs native ratios of 2102EP: (j) P53 PATHWAY (p < 0.001, q < 0.045), (k) HYPOXIA (p < 0.016, q < 0.145), (l) FATTY ACID METABOLISM (p < 0.042, q < 0.224), (m) GLYCOLYSIS (p < 0.006, q < 0.075), (n) ESTROGEN RESPONSE_LATE (p < 0.024, q < 0.116), (o) OXIDATIVE PHOSPHORYLATION (p < 0.011, q < 0.148) and (p) IL2 STAT5 SIGNALLING (p < 0.046, q < 0.133)

Supplementary file4 (PPTX 10786 KB) Supplementary Figure 3: Summary of the results of STRING analysis and DAVID annotation. (a, b) Increased proteins in NTERA-2-R cell lines: green – phosphoprotein, blue – acetylation, yellow – oxidoreductase, red – PcG protein complex. (c, d) Increased proteins in NCCIT-R cell lines: blue – oxidoreductase, red – mitochondrial biogenesis, green – cholesterol biosynthesis, yellow – lipid biosynthesis, pink – glutamine family amino acid biosynthetic process. (e, f) Increased proteins in 2102EP-R cell lines: blue – RNA N6-mehtyladenosine methyltransferase complex, red – glycogen storage disease. (g, h) Decreased proteins in NTERA-2-R cell lines: red – metabolism, green – phosphatidylinositol binding. (i, j) Downregulated proteins in NCCIT-R cell lines: red – metabolism, biosynthesis of amino acids, green – phosphatidylinositol binding. (k, l) Decreased proteins in 2102EP-R cell lines: green – Ubl conjugation, red – DNA methyltransferase activity, blue – microRNAs in cancer, yellow – kinase, pink – signal recognition particle

Supplementary file5 (PDF 1232 KB) Comparison of data to TCGA GCT cohort

## Data Availability

The mass spectrometry proteomics data have been deposited to the ProteomeXchange Consortium via the PRIDE [59] partner repository with the dataset identifier PXD030251. All other data is available on request from the corresponding author.
