# Supplementary figures and images for: Proteomic profiling of cisplatin-resistant and cisplatin-sensitive germ cell tumour cell lines using quantitative mass spectrometry
Source: World J Urol. 2022 Jan 27;40(2):373–83. doi: 10.1007/s00345-022-03936-1 (PMC8921118; doi:10.1007/s00345-022-03936-1)

## Slide 1
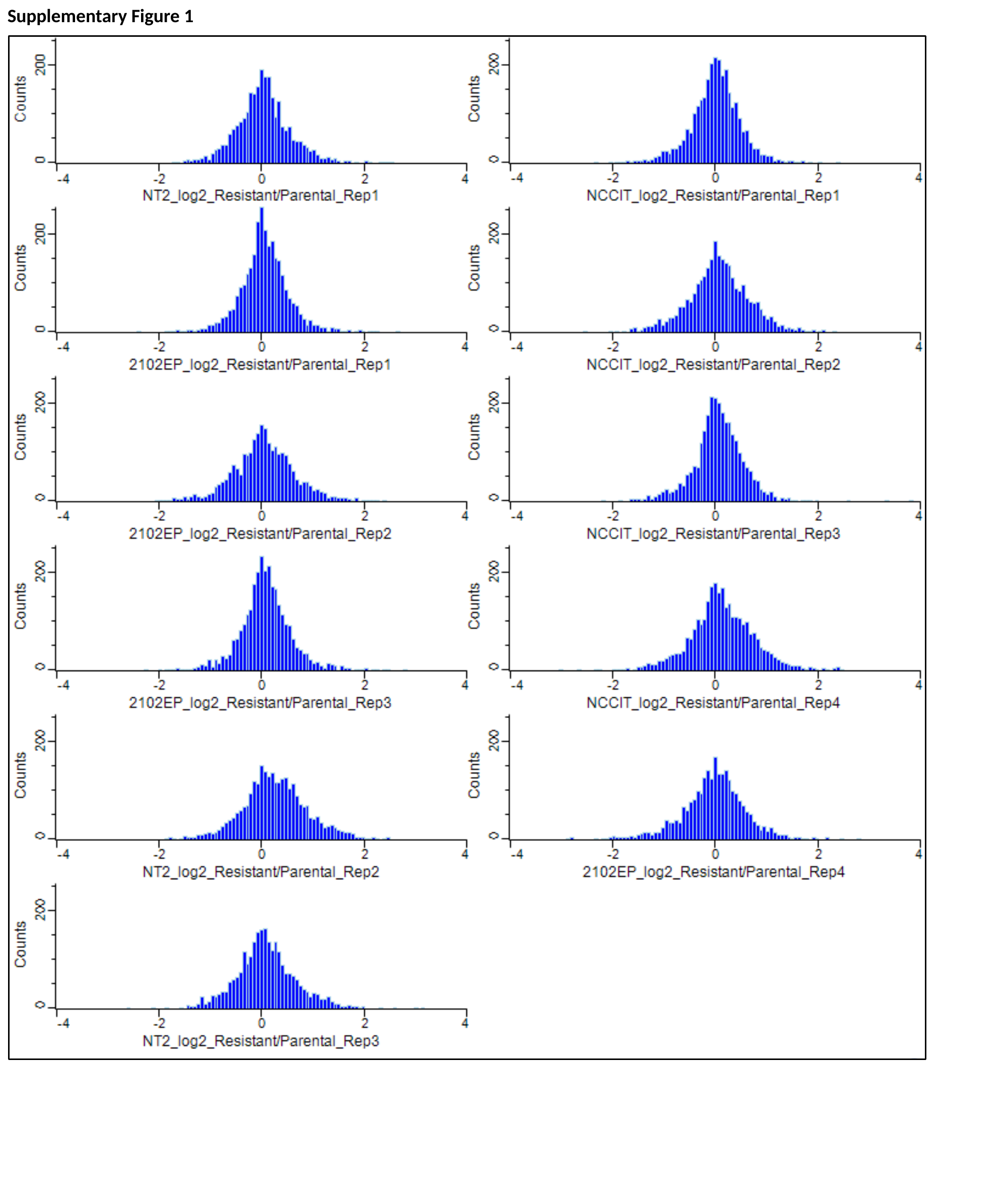

Supplementary Figure 1

Supplement: Supplementary file 1 — Supplementary file1 (PPTX 277 KB) Supplementary Figure 1: Distribution of proteins in all cell lines. This figure shows the normal distribution of proteins from SILAC analysis in all cisplatin-resistant and cisplatin-sensitive cell lines. [file 345_2022_3936_MOESM1_ESM.pptx]

## Slide 1
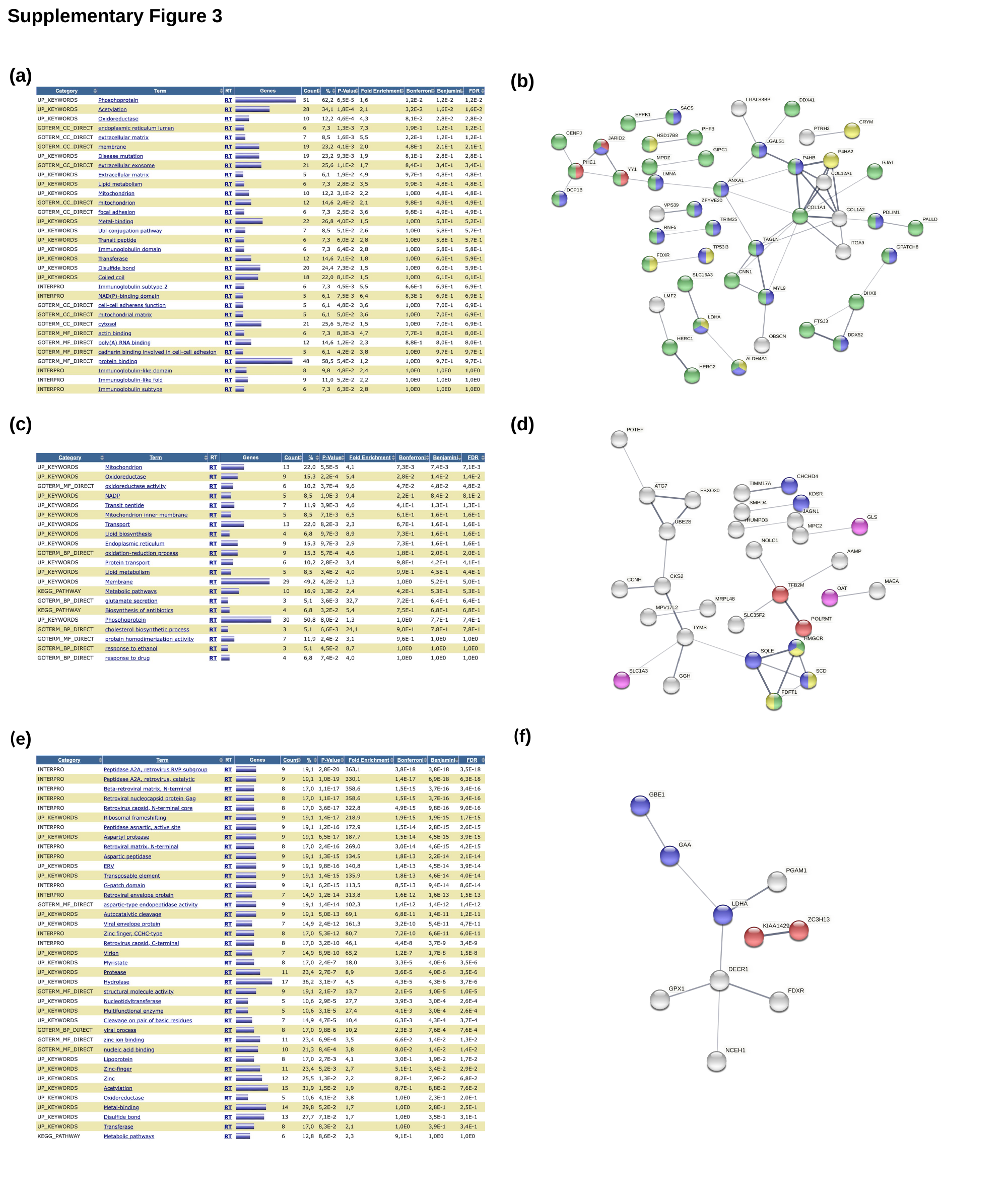

Supplementary Figure 3
(a)
(b)
(c)
(d)
(f)
(e)

## Slide 2
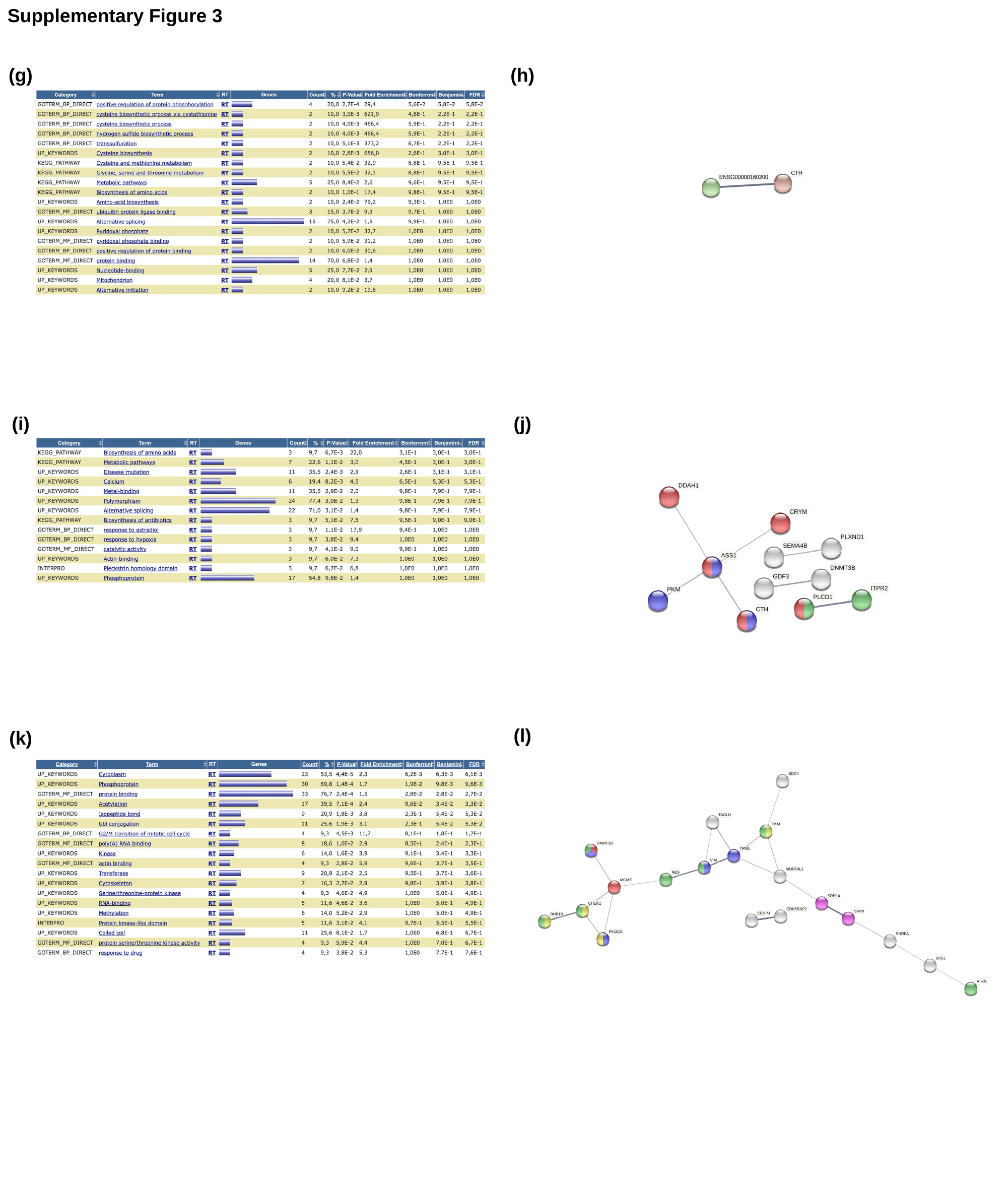

Supplementary Figure 3
(g)
(h)
(i)
(j)
(l)
(k)

Supplement: Supplementary file 4 — Supplementary file4 (PPTX 10786 KB) Supplementary Figure 3: Summary of the results of STRING analysis and DAVID annotation. (a, b) Increased proteins in NTERA-2-R cell lines: green – phosphoprotein, blue – acetylation, yellow – oxidoreductase, red – PcG protein complex. (c, d) Increased proteins in NCCIT-R cell lines: blue – oxidoreductase, red – mitochondrial biogenesis, green – cholesterol biosynthesis, yellow – lipid biosynthesis, pink – glutamine family amino acid biosynthetic process. (e, f) Increased proteins in 2102EP-R cell lines: blue – RNA N6-mehtyladenosine methyltransferase complex, red – glycogen storage disease. (g, h) Decreased proteins in NTERA-2-R cell lines: red – metabolism, green – phosphatidylinositol binding. (i, j) Downregulated proteins in NCCIT-R cell lines: red – metabolism, biosynthesis of amino acids, green – phosphatidylinositol binding. (k, l) Decreased proteins in 2102EP-R cell lines: green – Ubl conjugation, red – DNA methyltransferase activity, blue – microRNAs in cancer, yellow – kinase, pink – signal recognition particle [file 345_2022_3936_MOESM4_ESM.pptx]

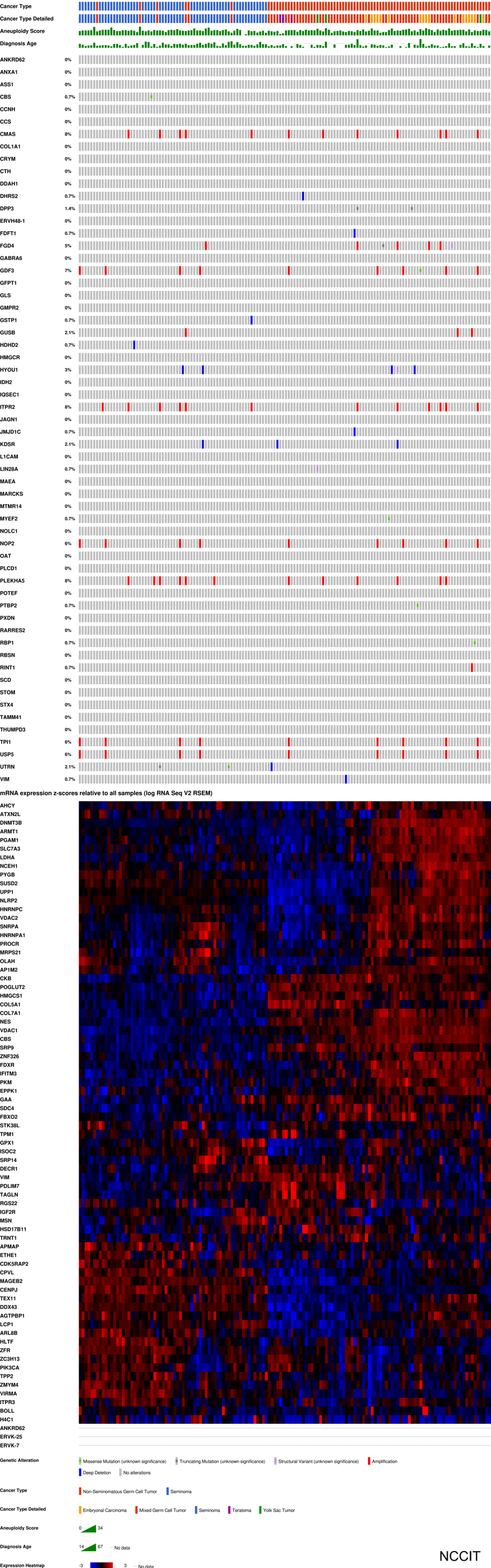

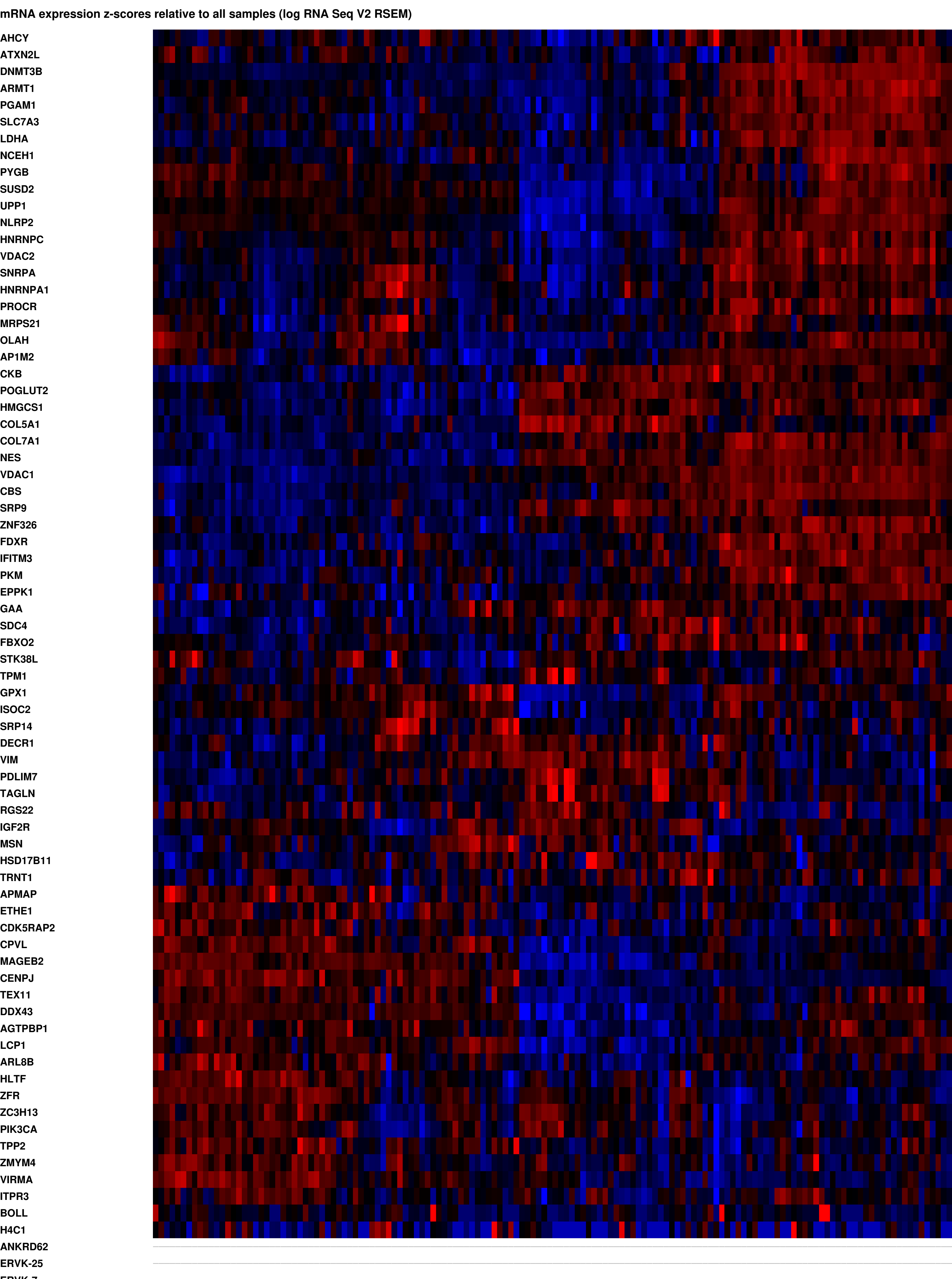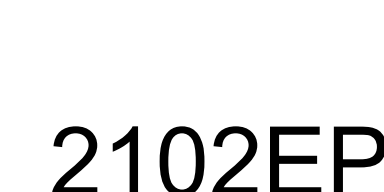

Supplement: Supplementary file 5 — Supplementary file5 (PDF 1232 KB) Comparison of data to TCGA GCT cohort [file 345_2022_3936_MOESM5_ESM.pdf]
