# Supplementary material for: Proteomic profiling of cisplatin-resistant and cisplatin-sensitive germ cell tumour cell lines using quantitative mass spectrometry
Source: World J Urol. 2022 Jan 27;40(2):373–83. doi: 10.1007/s00345-022-03936-1 (PMC8921118; doi:10.1007/s00345-022-03936-1)
Supplement: Supplementary file 3 — Supplementary file3 (PPTX 1041 KB) Supplementary Figure 2: GSEA analysis of all three GCT cell lines separated. (a) The top ten affected gene sets of NTERA-2 represented as the − log2 of the NOM q-value and the FDR q value. Significant enrichment plots of the resistant vs native ratios of NT2: (b) INTERFERON ALPHA RESPONSE (p < 0.001, q < 0.045), (c) INTERFERON GAMMA RESPONSE (p < 0.016, q < 0.145) and (d) MESENCHYMAL TRANSITION (p < 0.042, q < 0.224). (e) The top ten affected gene sets of NCCIT represented as the − log2 of the NOM q value and the FDR q value. Significant enrichment plots of the resistant vs native ratios of NCCIT: (f) MYC TARGETS_V1 (p < 0.000001, q < 0.014), (g) DNA REPAIR (p < 0.001, q < 0.096) and (h) MYC TARGETS V2 (p < 0.016, q < 0.111). (i) The ten top affected gene sets of 2102EP represented as the − log2 of the NOM q value and the FDR q value. Significant enrichment plots of the resistant vs native ratios of 2102EP: (j) P53 PATHWAY (p < 0.001, q < 0.045), (k) HYPOXIA (p < 0.016, q < 0.145), (l) FATTY ACID METABOLISM (p < 0.042, q < 0.224), (m) GLYCOLYSIS (p < 0.006, q < 0.075), (n) ESTROGEN RESPONSE_LATE (p < 0.024, q < 0.116), (o) OXIDATIVE PHOSPHORYLATION (p < 0.011, q < 0.148) and (p) IL2 STAT5 SIGNALLING (p < 0.046, q < 0.133) [file 345_2022_3936_MOESM3_ESM.pptx]

## Slide 1
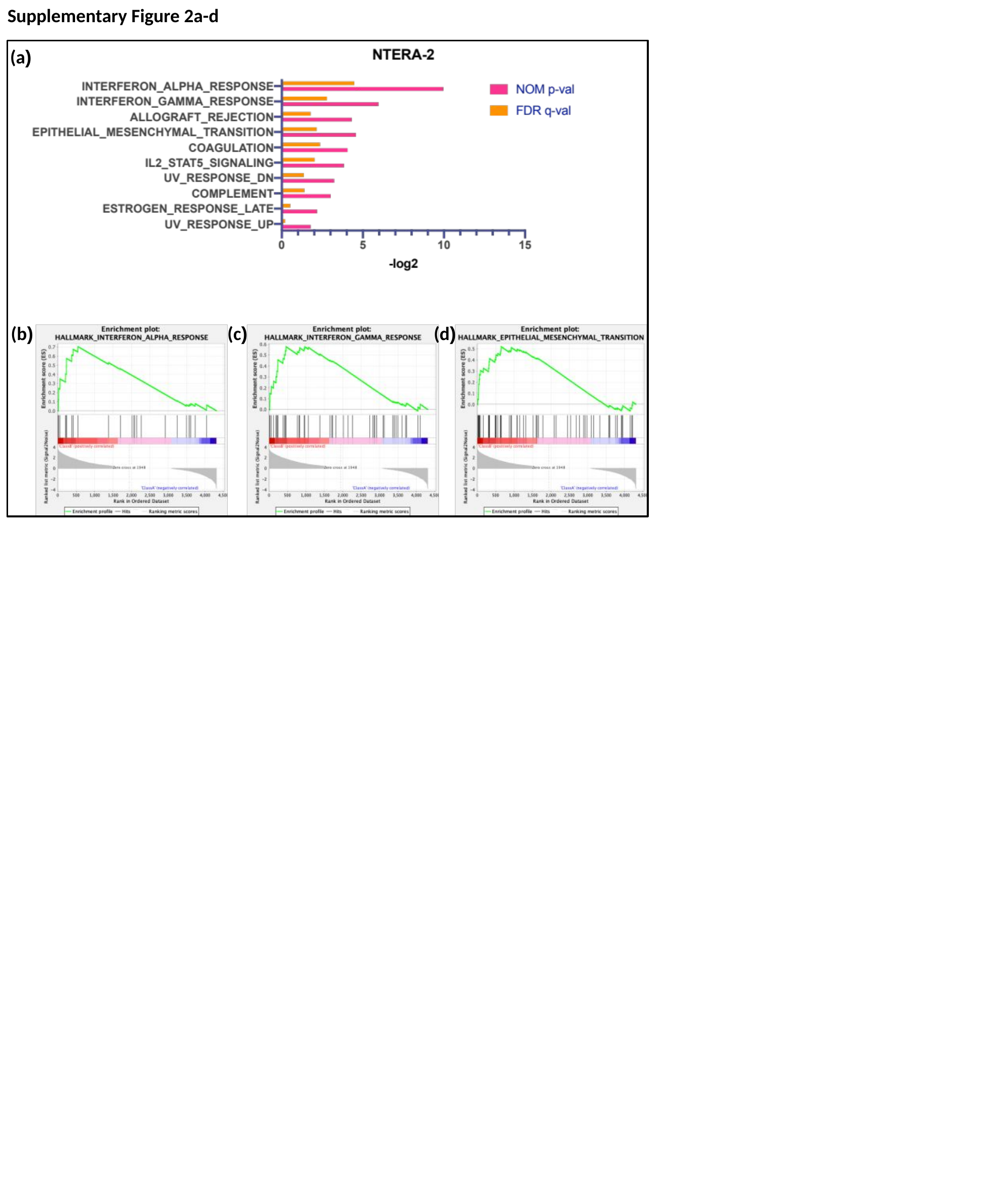

Supplementary Figure 2a-d
(a)
(b)
(c)
(d)

## Slide 2
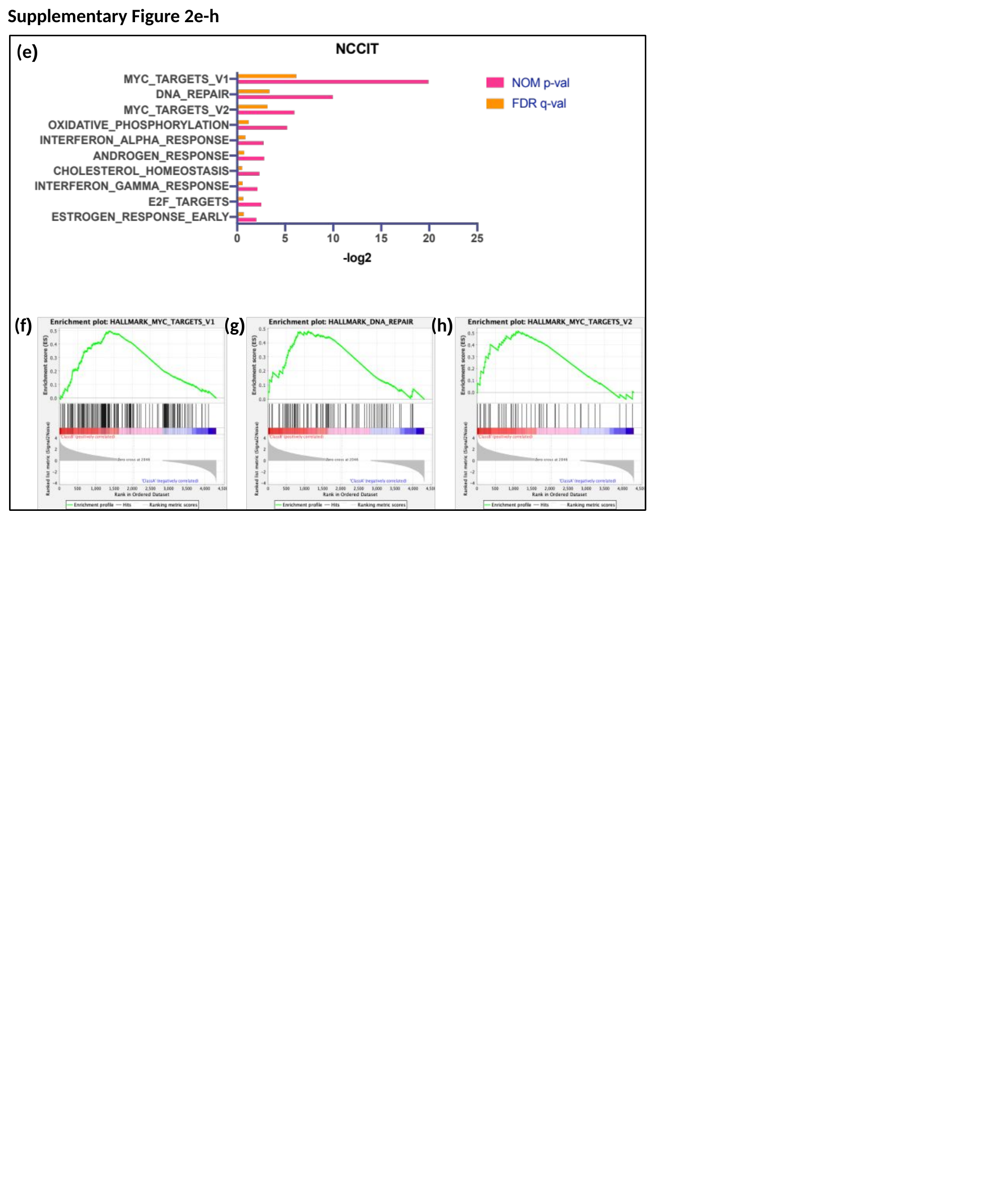

Supplementary Figure 2e-h
(e)
(f)
(g)
(h)

## Slide 3
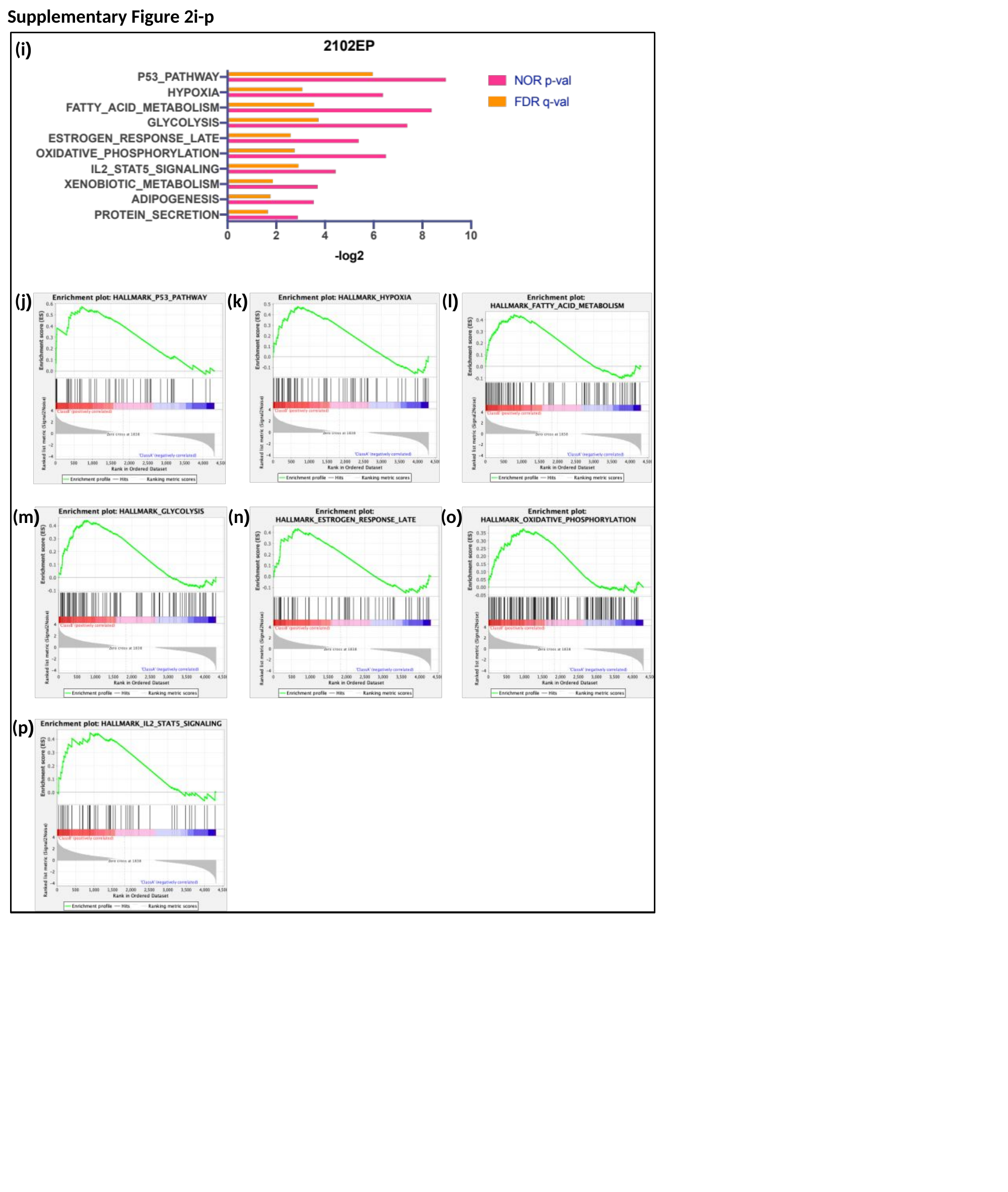

Supplementary Figure 2i-p
(i)
(j)
(k)
(l)
(m)
(n)
(o)
(p)
